# Sample Size Calculation in Dose Optimization Trials Using the Margin of Practical Non‐Inferiority

**DOI:** 10.1002/sim.70118

**Published:** 2025-05-19

**Authors:** Hakim‐Moulay Dehbi, Sean Devlins, Alexia Iasonos, Matthew Nankivell, Duncan Gilbert, John O'Quigley

**Affiliations:** ^1^ Comprehensive Clinical Trials Unit University College London London UK; ^2^ Memorial Sloan Kettering Cancer Centre New York NY USA; ^3^ MRC Clinical Trials Unit University College London London UK

**Keywords:** dose, dose optimization, dose reduction, randomized trial, sample size calculation

## Abstract

A dose optimization trial in oncology may be performed to compare an approved dose level of a given drug with a reduced dose level, testing the hypothesis that efficacy is maintained whilst reducing side effects and consequently improving adherence and quality‐of‐life. This is particularly relevant with modern therapeutic agents whose mechanisms of action imply that efficacy may not necessarily be linearly related to the dose. Using a conventional non‐inferiority framework leads to large sample sizes that are often unfeasible in the phase IV setting. An alternative is to use a margin of practical non‐inferiority, which we define in this paper and show how it can be exploited to justify a sample size. Whilst defining the extent of the margin, researchers also pre‐specify the other dimensions of interest, such as receptor occupancy and/or side effects and quality‐of‐life, that will be used to establish practical non‐inferiority if the observed efficacy of the reduced dose level lies within the margin. The comparison of efficacy is based on the observed difference between the reduced and the approved levels, instead of the confidence interval of this difference, leading to a reduction in sample size. The reduction in precision due to the smaller sample size is compensated by formally pre‐specifying the additional dimensions to the decision process, allowing a more thorough assessment of the opportunity to reduce a dose in practice, with the many advantages that this may involve.

## 1 Introduction

In recent years, there has been an increase in studies seeking to de‐escalate treatment, mostly in oncology but also in other medical areas [1]. These studies compare an approved dose level of a treatment to a lower dose level, testing the hypothesis that reduced treatment may be possible without compromising efficacy but with improvements in quality of life due to reduced toxicity, improved tolerability, and a potential reduction in costs. For example, the TROPIC trial demonstrated that prednisone combined with cabazitaxel at 25 mg/m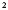 (C25) significantly improved overall survival (OS) compared to prednisone combined with mitoxantrone for men with metastatic castration‐resistant prostate cancer with progressive disease after docetaxel‐based treatment [2]. In the subsequent dose optimization trial, PROSELICA, a non‐inferiority design was chosen to demonstrate that a lower dose, cabazitaxel 20 mg/m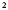 (C20), maintained at least 50% of the benefit seen with the approved dose (i.e., C25 against mitoxantrone in TROPIC). Non‐inferiority of C20 compared to C25 was confirmed for OS, but secondary endpoints indicated a more favorable clinical picture with C25 overall [3].

Dose reduction trials are a special case of dose optimization trials, which are carried out to fine‐tune the dose and schedule of previously licensed medical products. There are a number of considerations underlying dose optimization trials, including reducing toxicity, refining the methods of administration, or maximizing efficiency. Multiple dose optimization trials are currently ongoing, demonstrating an interest from patients, regulators, and, in general, the research community in dose optimization. Of note, the Optimal Cancer Care Alliance (OCCA), a charitable organization championing the principles of efficiency research, has identified at least ten cancer treatments where significant optimization of dosing schedules should be possible [4]. This is in keeping with the results of a review of the tolerability of molecularly‐targeted agents in phase III trials at doses and schedules recommended by phase I trials: 48% of patients required dose modification [5]. One of these ongoing trials is the MOIO trial that investigates a reduced dose intensity of immunotherapy by checkpoint inhibitors in responding patients with metastatic cancer [6]. Another example is REFINE, which looks at the effect of extended interval administration of immune checkpoint inhibitors on PFS in advanced cancers [7].

The mechanism of action of modern agents in oncology, such as targeted therapies or immunotherapy, is such that the dose‐efficacy curve may incorporate a plateau or be shallow [1, 8]. However, higher doses of the drug may result in increased short‐term toxicity and/or long‐term toxicity based on the total cumulative exposure, as has been shown with immune checkpoint inhibitors [9]. Given this context, less may be more in terms of drugs, as advocated by the FDA in Project OPTIMUS and other regulators [10, 11, 12, 13, 14]. Indeed, patients would experience fewer side effects, and consequently, their quality of life (QoL) may improve. Improved tolerability may allow patients to stay on the treatment longer. In addition, the cost of treatment could potentially decline, depending on the setting, which would be appealing [15, 16].

A recent case in point of dose optimization is the conditional approval received by Amgen from the FDA for sotorasib in May 2021. This drug is the first conditionally approved drug for metastatic KRAS G12C‐mutated non‐small‐cell lung cancer. The dose escalation study [17] did not demonstrate a clear dose‐effect relationship, with responses observed at the lower doses. However, the sample sizes at these doses were small. Moreover, PK parameters and plasma concentration time profiles were similar between dose levels. The FDA conditionally approved a dose of 960mg daily, whilst mandating a post‐approval comparison with 240mg (CodeBreak 201, ongoing).

A major design challenge of dose optimization trials is that the conventional non‐inferiority framework used in other settings typically requires large sample sizes, which may be difficult to achieve in phase IV trials. Novel study designs are required to answer the dosing questions of interest, with feasible sample sizes. The near‐equivalence notion has been introduced recently to identify cost‐effective treatments [18]. This framework articulates the different dimensions to consider, the main ones being efficacy, cost, pharmacokinetic (PK) and pharmacodynamic (PD) data, as well as QoL patient‐reported outcomes. To implement this idea in practice and justify a sample size, we propose to take a one‐sided version of the margin of practical equivalence [19, 20, 21], termed the margin of practical non‐inferiority. This idea builds upon prior research on practical equivalence in phase II selection trials. Recent trials have implemented the practical equivalence margin to identify a superior treatment amongst potentially active treatments without a control arm [22, 23, 24].

The practical non‐inferiority margin can incorporate QoL, PK/PD data, as well as cost considerations, into the decision‐making process to recommend a lower dose level as a replacement for the approved level. Dose optimization trials can be developed within this framework, leading to potentially lower sample sizes than if these were conventional non‐inferiority trials. The practical non‐inferiority margin can incorporate QoL, PK/PD data, as well as cost considerations, into the decision‐making process to recommend a lower dose level as a replacement for the approved level.

In this paper, we characterise this design strategy from a statistical perspective. We use the published results of TROPIC and PROSELICA to illustrate our proposal, which we also explore through simulation studies.

## 2 Dose Optimization Trial Design

### 2.1 Margin of Practical Non‐Inferiority

Before defining the margin of practical non‐inferiority, we recap on the margin of practical equivalence, which was initially developed for selection trials. These trials aim to select the most promising treatment option out of two (or more) options for further testing against a placebo or standard of care. Of note, there is no control arm in a selection trial. Sargent and Goldberg [19] suggested the implementation of a margin (of practical equivalence) to deal with situations where the difference in efficacy would not be sufficient to discriminate between the two options. If the two arms, in terms of observed median survival times, for example, are within the margin of practical equivalence, then other criteria (endpoints) apart from efficacy, such as QoL, tolerability, or cost, can be considered to make the selection. For example, the margin could be one month, which would mean that if the difference between the median survival times is less than 1 month, then the other pre‐specified criteria could be used to facilitate a selection. In two subsequent papers, Dehbi et al. developed this idea further for binary and time‐to‐event endpoints, and published online sample size calculators [20, 21].

**FIGURE 1:**
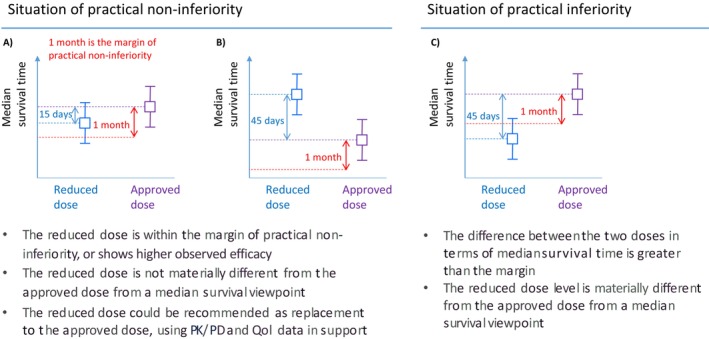
Overview of the possible scenarios in a dose reduction trial with a margin of practical non‐inferiority where the primary endpoint is a time‐to‐event endpoint such as overall survival or progression‐free survival. The squares correspond to the observed median survival times, and the whiskers correspond to their confidence intervals. (A) the reduced dose level and the approved dose level are within the margin of practical non‐inferiority based on the observed median survival times, (B) the reduced dose level shows greater efficacy than the approved dose level based on the observed median survival times, and (C) The reduced dose level is outside the margin of practical non‐inferiority based on the observed median survival times.

In a dose reduction trial, the margin of practical non‐inferiority is defined as the one‐sided equivalent of the margin of practical equivalence. If the observed difference in efficacy (e.g., median survival time) between the lower dose level and the approved level is less than the margin of practical non‐inferiority, then the reduced dose level could be considered not materially different from the approved dose. This assessment would be complemented by QoL as well as PK/PD data, and possibly other pre‐specified endpoints. If the totality of the data is favoring practical non‐inferiority, then the reduced dose level could be recommended as an alternative to the approved level. Figure 1 describes the possible situations at study completion in such a trial if a margin of practical non‐inferiority is defined. We use 1 month for the margin for illustrative purposes.

### 2.2 Sample Size Calculation

#### 2.2.1 Statistical Framework

In statistical terms, let $P_{non-inf}$ and $P_{inf}$ denote the probabilities of the two states described in Figure 1 (i.e., practical non‐inferiority or practical inferiority) across hypothetical repetitions of the trial, with $P_{non-inf}+P_{inf}=1$. $P_{non-inf}$ refers to the possibility that the lower dose level will be within the margin of practical non‐inferiority. In other words, the difference in observed median survival times between the doses is less than the margin. It might be that the difference is in favor of the reduced dose, also corresponding to a situation of practical non‐equivalence. The objective is then to derive a sample size such that there is a chance that exceeds a certain predefined threshold Q (e.g., 80%) that the study reaches a situation of practical non‐inferiority, assuming that the two dose levels provide the same efficacy on the efficacy endpoint. Note that this assumption is also the starting point of a conventional non‐inferiority sample size calculation. However, a second perspective must be considered. One must ensure that if the reduced dose level is equivalent to the control arm of the previous phase III confirmatory trial, then there is a chance that exceeds a certain pre‐specified threshold Z that the study concludes that the reduced dose level is outside the margin and consequently perceived as practically inferior.

#### 2.2.2 Calculation for a Time‐To‐Event Endpoint

Consider two dosage levels for simplicity, denoted as levels 1 and 2. Dose level 1 is the reduced dose level, and dose level 2 is the approved dose level. Similar to many power calculations in clinical trials with a time‐to‐event endpoint, let us assume that the survival times T are exponentially distributed, that is, $T_{1}∼\mathrm{Exp}\;(\lambda_{1})$ and $T_{2}∼\mathrm{Exp}\;(\lambda_{2})$, $\lambda_{i}>0$, i=1,2, with exponential density $f(t)=\lambda e^{-\lambda t},t>0$. Based on the published median survival time of the approved dose level ${\tilde{T}}_{\text{approved}}$ from the phase III trial, the point estimate for $\lambda_{2}$ is $\ln(2)/{\tilde{T}}_{\text{approved}}$.

In the common case of survival data with non‐informative right censoring, the sampling distribution of $\ln\hat{\lambda }$ is normally distributed [21]. It follows that the distribution of $\ln\hat{\lambda_{2}}-\ln\hat{\lambda_{1}}$ is also normally distributed, and more precisely that $\ln\hat{\lambda_{2}}-\ln\hat{\lambda_{1}}∼N(\ln\lambda_{2}-\ln\lambda_{1},\frac{2}{np})$ where n is the sample size per arm and p is the probability of non‐censoring for the individual observations. Given that the ratio $\frac{\lambda_{2}}{\lambda_{1}}$ is the hazard ratio (HR) in this case, the previous expression reduces to $\ln\hat{\lambda_{2}}-\ln\hat{\lambda_{1}}∼N(\ln(\text{HR}),\frac{2}{np})$. Consequently, the required sample size can be calculated by making use of the Gaussian distribution.

For sample size calculation purposes, we must consider the following two perspectives: Firstly, that the reduced dose level is equivalent to the current approved level efficacy‐wise, and secondly, that the reduced dose level is not any better than the previous phase III control treatment (or some other meaningful decrease in median survival).

The first perspective is articulated as follows. We assume that $\lambda_{1}=\lambda_{2}$, which implies that the median survival time with dose 1 is equal to that with dose 2, that is, $\tilde{T_{1}}=\ln(2)/\lambda_{1}=\tilde{T_{2}}=\ln(2)/\lambda_{2}$. By assuming $\lambda_{1}=\lambda_{2}$ we effectively imply that $\ln\hat{\lambda_{2}}-\ln\hat{\lambda_{1}}∼N(\ln(\text{1})=0,\frac{2}{np})$. Denoting $P_{non-inf}$ as π, we express the dependency on n as:

$$\pi_{n}({\text{MPE}}_{non-inf})=\mathrm{Pr}\;({\text{MPE}}_{non-inf}\leq \ln\hat{\lambda_{2}}-\ln\hat{\lambda_{1}})$$

where ${\text{MPE}}_{non-inf}=\ln({\text{HR}}_{non-inf})$. This simple expression is derived from the fact that $\frac{\lambda_{2}}{\lambda_{1}}=\frac{\ln(2)/{\tilde{T}}_{\lambda_{2}}}{\ln(2)/{\tilde{T}}_{\lambda_{1}}}=\frac{{\tilde{T}}_{\lambda_{1}}}{{\tilde{T}}_{\lambda_{2}}}$. ${\text{HR}}_{non-inf}$ is thus the margin of practical non‐inferiority in terms of the ratio of medians. For example, ${\text{HR}}_{non-inf}=0.90$ would indicate that the margin of practical non‐inferiority is a ratio of medians greater than or equal to 90%. Based on the Gaussian distribution and setting the threshold to Q (e.g., 80%) we then calculate the sample size required such that:

$$\begin{array}{l} {\hat{n}}_{non-inf}({\text{MPE}}_{non-inf},Q)=\arg\min\left\{n\in \mathbb{N}:\pi_{n}({\text{MPE}}_{non-inf})\geq Q\right\} \end{array}$$

It is not known for certain that $\lambda_{1}=\lambda_{2}$. This is what the second perspective refers to. It could be that the additional benefit demonstrated by the approved dose level above the control arm observed in a previous phase III trial is not maintained by the reduced dose level. This possibility can be quantified. Assuming that the historical control arm median survival time ${\tilde{T}}_{\text{control}}$ corresponds to the median survival of the reduced dose level, that is, $\lambda_{1}=\ln(2)/{\tilde{T}}_{\text{control}}$, one can derive the probability that the trial will not conclude with practical non‐inferiority, which is desirable in this case. In statistical terms, denoting the dependency on n of $P_{inf}$ by $\kappa_{n}$ we calculate $\kappa_{n}({\text{MPE}}_{non-inf})=\mathrm{Pr}\;(\ln\hat{\lambda_{2}}-\ln\hat{\lambda_{1}}<{\text{MPE}}_{non-inf})$. The required sample size with respect to Z, which is a predefined threshold (e.g., 80%), is then:

$${\hat{n}}_{inf}({\text{MPE}}_{non-inf},Z)=\arg\min\left\{n\in \mathbb{N}:\kappa_{n}({\text{MPE}}_{non-inf})\geq Z\right\}$$

The recommended sample size for the study, as a function of Q and Z, is $\max({\hat{n}}_{non-inf},{\hat{n}}_{inf})$.

Traditional frequentist sample size calculations for time‐to‐event endpoints are expressed in terms of a number of events for a given power. In the context of the margin of practical non‐inferiority, we calculate a number of patients such that the probability is, for example, 80% (or 90%) that the difference in the observed medians is of a certain magnitude. The proportion of patients experiencing the event, or being censored, is taken into account via the parameter p that influences the sampling distribution of $\ln\hat{\lambda_{2}}-\ln\hat{\lambda_{1}}$.

#### 2.2.3 Calculation for a Binary Endpoint

The same statistical framework is used to calculate the required sample size when the primary endpoint is binary. This time, the margin of practical non‐inferiority is defined as a difference in the percentage of success between the two dose levels (e.g., difference in objective response rates).

Exact binomial probabilities, or Monte Carlo simulations, can derive the sample size required so that $P_{non-inf}$ is above a predefined threshold Q (e.g., 80%) if the two dose levels are equivalent. This is the equivalent of the first perspective referred to above. As with time‐to‐event endpoints, if the reduced dose level is not any better than the control arm of the previous phase III trial or a predefined meaningful decrease in the outcome, the probability of not concluding practical non‐inferiority can also be calculated using the exact binomial distribution or Monte Carlo simulations. One must ensure that this probability is greater than a certain predefined threshold Z (e.g., 80%), which is the second perspective to take into account in the sample size calculation.

The notations are the following. For dose level 1, which is the reduced dose level, $n_{1}∼Bin(n,p_{1})$ where $n_{1}$ is the number of successes (e.g., number of responders in a cancer clinical trial, or number of patients alive and progression‐free) out of n participants, and $p_{1}$ is the probability of success. Similarly, for dose level 2, which is the approved dose level, $n_{2}∼Bin(n,p_{2})$.

For the first perspective, we assume that $p_{1}=p_{2}$. Denoting $P_{non-inf}$ as π, we express the dependency on n as:

$$\pi_{n}({\text{MPE}}_{non-inf})=\mathrm{Pr}\;(\hat{p_{2}}-\hat{p_{1}}\leq {\text{MPE}}_{non-inf})$$

${\text{MPE}}_{non-inf}$ is defined in terms of percentage points. For example, ${\text{MPE}}_{non-inf}=0.05$ means that a difference of 5 percentage points or less between the approved dose level and the reduced dose level, in terms of percentage of success, would mean practical non‐inferiority. Using a grid search, the sample size that is required so that the probability of practical non‐inferiority is larger than the predefined cut‐off Q is:

$$\begin{array}{l} {\hat{n}}_{non-inf}({\text{MPE}}_{non-inf},Q)=\arg\min\left\{n\in \mathbb{N}:\pi_{n}({\text{MPE}}_{non-inf})\geq Q\right\} \end{array}$$

For the second perspective, we assume that $p_{2}$ is equal to $p_{experimental}$, which is the observed response rate in the experimental arm of the preceding phase III trial, and that $p_{1}$ is equal to $p_{control}$ (which is the response rate of the control arm in the previous trial).

As with time‐to‐event endpoints, we denote the dependency on n of $P_{inf}$ by $\kappa_{n}$ and calculate $\kappa_{n}({\text{MPE}}_{non-inf})=\mathrm{Pr}\;(\hat{p_{2}}-\hat{p_{1}}>{\text{MPE}}_{non-inf})$. The required sample size with respect to Z, which is a predefined threshold (e.g., 80%), is found using a grid search:

$${\hat{n}}_{inf}({\text{MPE}}_{non-inf},Z)=\arg\min\left\{n\in \mathbb{N}:\kappa_{n}({\text{MPE}}_{non-inf})\geq Z\right\}$$

The recommended sample size for the study, as a function of Q and Z, is $\max({\hat{n}}_{non-inf},{\hat{n}}_{inf})$.

## 3 Case Study: TROPIC and PROSELICA Trials

**FIGURE 2:**
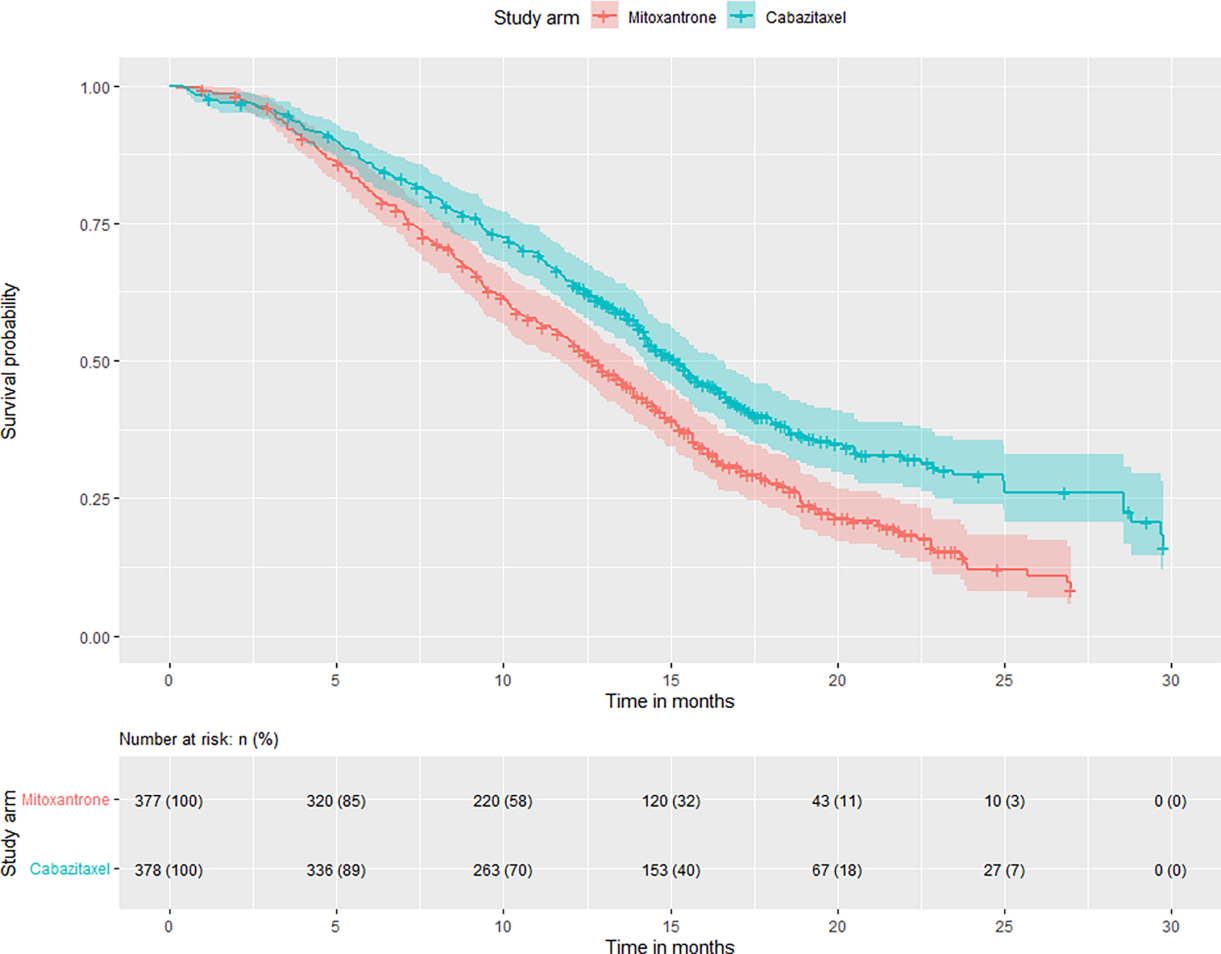
Kaplan‐Meier survival curves in TROPIC, based on reconstructed individual patient data.

The last decade has seen major progress in the treatment of men with advanced prostate cancer, a situation where, until relatively recently, the only effective treatment was androgen deprivation. TROPIC [2] compared the chemotherapy drug Cabazitaxel (at a dose of 25mg/m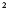 every 3 weeks‐C25) with Mitoxantrone in men with progressive disease after first‐line treatment (Docetaxel). It demonstrated an improved median survival time compared to Mitoxantrone from 12.7 months (95% confidence interval (CI): 11.6–13.7 months) to 15.1 months (95% CI: 14.1–16.3 months) for men with metastatic castration‐resistant prostate cancer with progressive disease after Docetaxel‐based treatment. In Figure 2, the Kaplan‐Meier survival curves are presented based on the reconstructed individual patient data from the TROPIC published data [25]. Concerns around the toxicity seen at this dose of Cabazitaxel led to the PROSELICA trial, which subsequently compared a lower dose (20mg/m
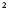
‐C20) of Cabazitaxel with the dose obtained from the TROPIC trial, with the primary outcome measure of overall survival.

In this case study, we define the margin of practical non‐inferiority at 90% for C20 treatment (the experimental arm in PROSELICA). Assuming that the median survival time in the dose optimization study is 15.1 months again for C25 treatment, then if the median survival time for C20 is ≥13.6, C20 would be practically non‐inferior to C25, at least in terms of OS. The margin of practical non‐inferiority is 1.5 months effectively.

**FIGURE 3:**
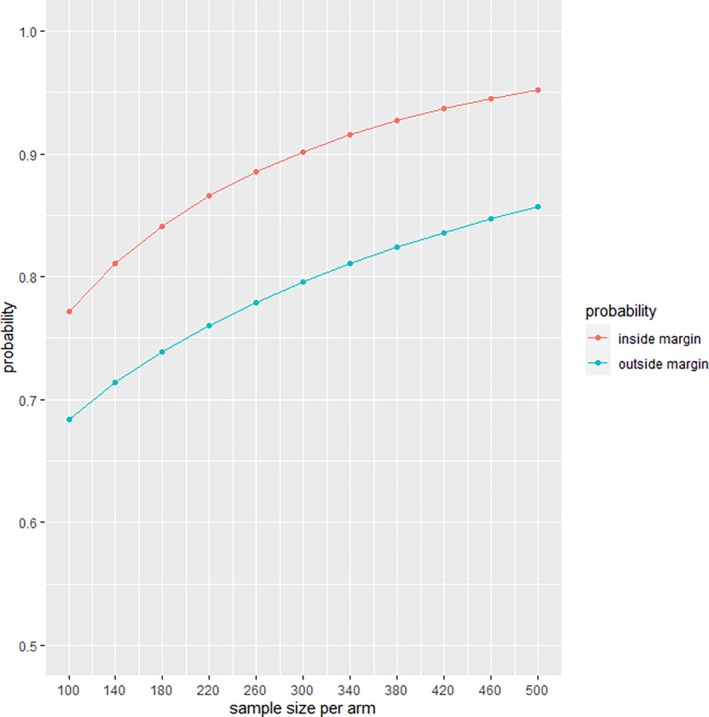
Probability $\kappa_{n}({\text{MPE}}_{non-inf})$ to be outside the margin at study end if the reduced dose arm C20 is equivalent to mitoxantrone, and probability $\pi_{n}({\text{MPE}}_{non-inf})$ to be inside the margin at study end, if C20 is equal to C25 in terms of median survival time.

Figure 3 reports ${\hat{n}}_{non-inf}$ and ${\hat{n}}_{inf}$ using the median OS times in TROPIC, with an assumed probability of censoring of 0% just for illustration purposes. A sample size of 300 patients per arm provides a 90% chance to achieve practical non‐inferiority for C20, assuming that C20 is equivalent to C25 on overall survival. This sample size also provides an 80% chance that the observed medians are not within the margin if indeed C20 is actually equivalent to mitoxantrone, in other words, that it does not maintain the benefit demonstrated by C25 over the control arm of TROPIC. The assumption of 0% censoring is for illustrative purposes. In an actual trial, this percentage would be based on the best estimate of the proportion of patients expected to be censored.

**FIGURE 4:**
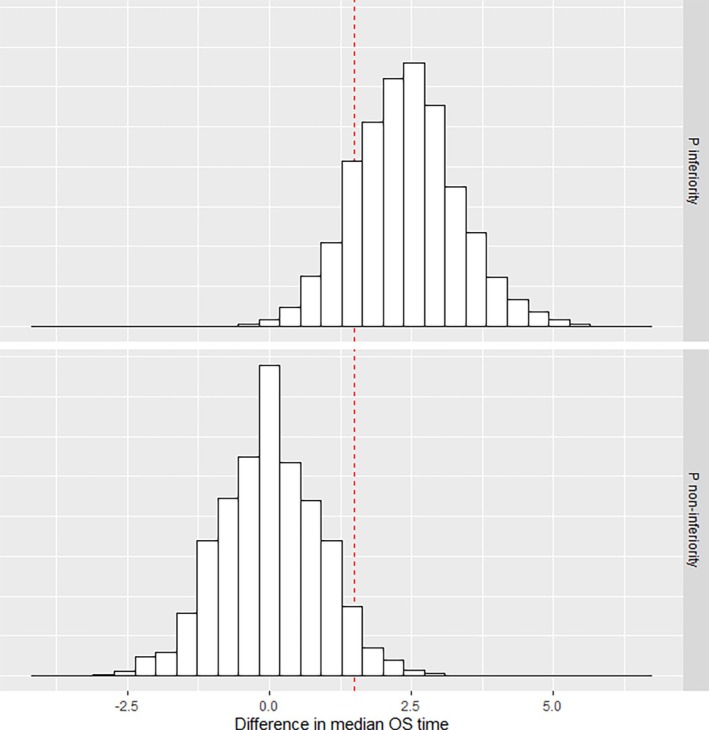
Histograms of observed differences between median survival times in 10 000 replicates based on the reconstructed individual patient data from TROPIC. The vertical red line denotes the margin of practical non‐inferiority of 1.5 months.

We verified this sample size calculation using the reconstructed individual patient data from the TROPIC published data. This is indeed the data researchers designing PROSELICA would have had at the time. Using 10 000 replicates, based on a sample size of 300 patients per arm obtained from sampling with replacement from the experimental and control arms of TROPIC, we calculated $P_{non-inf}$ and $P_{inf}$. The histograms of the observed differences between median survival times are shown in Figure 4, which indicates $P_{non-inf}$ is 96% and $P_{inf}$ is 84% chance. These percentages are slightly higher than the calculated percentages, which is due to the fact that the exponential distribution used for calculation is only an approximation to the observed data. In any given scenario, if the phase III data are available, verifying the calculated sample size using a Monte‐Carlo approach (incorporating sampling with replacement) is recommended. In this specific case study, the data is such that the calculated sample size underestimates $P_{non-inf}$.

The sample size of 600 patients in total represents half the sample size of PROSELICA, which was designed using a conventional non‐inferiority margin. In PROSELICA, the median OS times were 13.4 months (95% confidence interval (CI): 12.2–14.9 months) for C20 and 14.5 months (95% CI: 13.5–15.3) for C25. This difference of 1.1 months is less than the margin that we defined in this illustration.

## 4 Discussion

In a dose reduction trial, we must avoid potentially exposing a large number of patients to a lower dose level that may not maintain the demonstrated benefit of the approved level. Designing dose optimization trials with the best interests of patients in mind is paramount. Conventional non‐inferiority designs require a large number of patients, except if the margin of non‐inferiority is markedly relaxed, which defeats the purpose. By using a margin of practical non‐inferiority, supplemented by other endpoints such as receptor occupancy for targeted agents, PK/PD, and QoL data, one can assess in a structured way the clinical picture of a reduced dose level with a modest sample size compared to a full‐scale, conventionally defined non‐inferiority study. The saving in sample size comes from the fact that instead of looking at the upper end of the confidence interval for the treatment effect (most commonly estimated by a hazard ratio or a difference in proportions surviving up to a fixed time point), one considers the observed difference in proportions or median survival times. The other dimensions that are pre‐specified alongside the margin of practical non‐inferiority, in particular PK/PD and QoL data, provide an opportunity to assess the reduced dose level in a comprehensive manner, instead of focusing solely on one endpoint. A real strength of the margin of practical non‐inferiority is that it is expressed on the original unit of survival time, compared to the conventional non‐inferiority margin that is expressed on a hazard ratio, which is less easily interpretable [26].

An understanding of the mechanism of action of modern agents is particularly important in modern oncology dose reduction trials and, hence, a move to consider biological or functional outcome measures. For immune checkpoint inhibitors, for example, using PD‐1, measurement of PD‐1 T‐cell receptor occupancy (one indication of how effective the drug is) could be one of the dimensions considered to determine practical non‐inferiority. In a scenario where the reduced dose level provides similar occupancy, is associated with improved QoL, and if the observed difference in median survival times (or proportions surviving past a certain time point) is within the predefined boundary, then it is conceivable that the lower dose level could be recommended as an alternative to the approved level. This would, in turn, lead to major savings in time over a non‐inferiority study, potential money for patients and healthcare systems, and importantly, reducing long‐term side effects and improved tolerability.

Setting the margin of practical non‐inferiority should be done by combining qualitative inputs, including the views of multiple stakeholders, especially patients, along with quantitative inputs. From a quantitative viewpoint, the starting point should be the demonstrated benefit of the approved dose level. In TROPIC, for example, a gain of 2.4 months in median survival time was observed for C25 against mitoxantrone. Reasonable starting values for the margin would be 25% or 33% of this difference, assuming other meaningful benefits for the reduced dose arm are also seen. Additionally, using simulations, one could identify the length of the practical non‐inferiority margin such that, for a given sample size, the conditional probability of achieving conventional non‐inferiority, if practical non‐inferiority is met, is above a certain threshold.

In the time‐to‐event setting, we have designed the margin of practical non‐inferiority based on median survival. This will likely work for many clinical scenarios. However, median survival may not be reached in some settings, such as early‐stage disease. In such settings, the methodology can be easily extended to a survival probability at a fixed time point, such as one year following randomization. Under this framework, the study would examine the difference between the 1‐year survival probability of the approved dose and the reduced dose.

We exploit the exponential distribution to derive the required sample size in our proposed approach. It is a common and useful approximation in time‐to‐event sample size calculations. However, dose optimization trials take place after large phase III trials are completed. This means that researchers might be able to obtain individual patient data, or digitize published Kaplan‐Meier curves to verify the calculation based on the actual data distribution of the control and experimental arms of the previous phase III data. We strongly encourage researchers to use Monte‐Carlo simulations to ensure that the sample size calculation is robust to departures from the exponential data assumption.

In summary, we propose using the margin of practical non‐inferiority for dose reduction trials. These trials are in the best interest of patients and may demonstrate that the reduced dose levels maintain efficacy and reduce side effects, which in turn would improve adherence, retention, and potentially clinical outcomes. In addition, healthcare systems could make some much‐needed savings to reinvest elsewhere, in research or clinical care.

## Disclosure

The authors have nothing to report.

## Conflicts of Interest

The authors declare no conflicts of interest.

## Data Availability

Data sharing is not applicable to this article as no new data were obtained or analyzed in this study. We only used simulated data.
